# Clinicopathologic Parameters of Peritoneal Fluid as Predictors of Gastrointestinal Lesions, Complications, and Outcomes in Equine Colic Patients: A Retrospective Study

**DOI:** 10.3390/ani15010012

**Published:** 2024-12-24

**Authors:** Emily Martin, Kate Sarkan, Austin Viall, Shannon Hostetter, Kira Epstein

**Affiliations:** 1 Department of Clinical Sciences, North Carolina State University College of Veterinary Medicine, Raleigh, NC 27606, USA; egmedlin@ncsu.edu; 2 Department of Veterinary Biosciences, The Ohio State University College of Veterinary Medicine, Columbus, OH 43210, USA; sarkan.2@buckeyemail.osu.edu; 3 Department of Pathology, Microbiology, and Immunology, University of California Davis School of Veterinary Medicine, Davis, CA 95616, USA; akviall@ucdavis.edu; 4 Department of Pathology, University of Georgia College of Veterinary Medicine, Athens, GA 30602, USA; shannon.hostetter@uga.edu; 5 Department of Large Animal Medicine, University of Georgia College of Veterinary Medicine, Athens, GA 30602, USA

**Keywords:** equine, peritoneal fluid, neutrophil, colic, gastric reflux, SIRS

## Abstract

Neutrophil characteristics in peritoneal fluid (PF) may aid in diagnosing and treating specific colic lesions and complications. The objective of this retrospective study was to evaluate quantitative PF leukocyte values, as well as PF total protein (TP) and lactate, for associations with diagnosis, morbidity, and mortality in horses with acute colic. Three hundred and forty-two horses that presented to one institution between January 2010–2020 for the evaluation of acute colic were included. The PF total nucleated cell count (TNCC), % and total neutrophil counts, total protein (TP), and lactate were analyzed for associations with lesion location and type, the development of postoperative reflux (POR) or systemic inflammatory response syndrome (SIRS), and survival to discharge via Kruskal–Wallis testing. Horses with strangulating lesions had higher PF % neutrophils, neutrophil count, and TNCC compared to non-strangulating lesions. The development of SIRS or POR was associated with higher PF TNCC, total neutrophil count, TP, and lactate. Horses that did not survive to discharge had increased PF % neutrophils, neutrophil count, TP, lactate, and ratio of PF-to-systemic TP than those that survived via univariable analysis. Identified associations between increased PF neutrophils and the development of POR and SIRS warrant further investigation to better understand their role in the pathogenesis of equine colic and potential as targets for therapeutic intervention.

## Simple Summary

Delays in diagnosis and treatment of equine colic can lead to poor outcomes. The analysis of fluid from the abdominal (or “peritoneal”) cavity is a valuable means for assessing peritoneal and intestinal health. Specific peritoneal fluid biomarkers can be evaluated to rapidly identify gastrointestinal lesions that require surgery. However, some colic cases are not straightforward, and thus additional biomarkers are needed. Neutrophils are white blood cells that respond quickly to infection and tissue damage and can increase in peritoneal fluid during colic. In this study, we retrospectively evaluated neutrophil counts in equine peritoneal fluid of colic horses to understand if they can help us to predict the strangulated nature of a gastrointestinal lesion and potential outcomes. Horses with strangulating gastrointestinal lesions, which occur when blood supply has been lost to the tissues and requires surgery, had higher peritoneal fluid neutrophils than horses that did not have strangulating lesions. Additionally, horses that developed complications and those that did not survive to discharge also had higher peritoneal fluid neutrophil counts. In conclusion, peritoneal fluid neutrophil counts may help to predict gastrointestinal lesion types and outcomes, and the role of neutrophils in these gastrointestinal lesions and complications warrants further study to understand if they might be a target for therapies.

## 1. Introduction

Abdominocentesis, or the collection of free peritoneal fluid (PF) from the abdominal cavity, is a common diagnostic tool used in equine practice to aid in determining the cause of colic. The analysis of PF can provide a representative sample from within the abdomen to assess gastrointestinal (GI) health. When interpreted in conjunction with other clinical parameters, PF analysis can aid in reaching the appropriate diagnosis, implementing treatment, and monitoring disease progression more rapidly. Changes in PF parameters can manifest within 1–2 h of onset in cases of ischemic GI lesions [1], which are generally more rapid than alterations to systemic blood parameters. Lesions causing the ischemia of GI tissue invariably require surgical correction, and addressing these lesions sooner may minimize systemic compromise, negate the need for intestinal resection, and improve prognosis. In addition to the benefits of early diagnosis, abdominocentesis is a relatively straightforward procedure with a low risk of morbidity in horses [2] and a high yield of information.

Common clinicopathologic values analyzed in equine PF include white blood cell count and differential, total protein concentration, lactate concentration, glucose concentration, and a cytological evaluation for cellular morphology and the presence of microorganisms [3]. Gross fluid characteristics, including color and turbidity, are also routinely included in the fluid analysis. No single parameter has been identified that reliably differentiates between different causes of colic, and alterations in these values outside of normal reference ranges must be interpreted in light of other clinical examination and systemic clinicopathologic findings. Frequently, even in combination, not all findings clearly point to a single diagnosis or even diagnostic group, resulting in challenges to equine practitioners trying to determine the best course of action for a given horse with colic. Thus, there is a need to continue to expand our understanding of PF clinicopathology and improve diagnostic accuracy.

The major cell type found within equine PF in both health and disease is the neutrophil. Nondegenerate neutrophils normally comprise between 40 and 80% of the total nucleated cells within the fluid and are present at a 2:1 ratio to mononuclear cells [3]. Neutrophils play a dual role in health and disease in that they provide a critical first line of defense against microorganisms but can also exert collateral damage to host tissues when mobilized to combat infection or disease. Neutrophils accumulate within reperfused GI tissue following periods of ischemia [4], where they perpetuate inflammation and elicit substantial tissue damage through dysregulated cytokine, reactive oxygen species (ROS), and neutrophil extracellular trap (NET) production [5]. Neutrophils also play a pivotal role in the development of systemic inflammatory response syndrome (SIRS), which is a common sequela of GI inflammation in horses [6], through the production of proinflammatory cytokines, direct endothelial damage, and vascular occlusion via the production of ROS and NETs. These events can lead to tissue ischemia and multiple organ dysfunction (MODs). Similarly, GI ileus has been proposed to be linked to inflammation within GI tissues, including a massive influx of neutrophils following bowel manipulation during surgery [7] that contribute to tissue damage, inflammation, and edema that may cause decreased motility [8]. Ileus commonly presents in horses as GI reflux, as ingesta is unable to be transported aborally by normal propulsive mechanisms due to intestinal stasis.

Despite the possible role of neutrophils in both SIRS and ileus in colic patients, research to determine associations between PF neutrophils, the development of these complications, and survival is limited. Additionally, their diagnostic utility within PF to differentiate between causes of colic has not been thoroughly established. Previous studies have shown that while PF total nucleated cell count did not differ between types of colic [2,9], intestinal ischemia can lead to an increase in the ratio of neutrophils to mononuclear cells, and the concentration and % neutrophils within the PF to upwards of 50,000–150,000 cells/μL and 90–95%, respectively [1]. However, these changes can also be seen in non-ischemic tissue lesions, depending on the time at which the sample is collected and the severity of the lesion. Thus, these values must be considered in light of other PF and systemic parameters to interpret their association with specific lesion types, the development of complications, and survival. However, these relationships have not been thoroughly established and leave room for further exploration.

The aim of this retrospective study was to evaluate quantitative PF neutrophil values including absolute values, the percentage of total PF nucleated cell counts, and ratios to systemic quantitative neutrophil values, for associations with colic diagnosis, morbidity, and mortality in horses with acute signs of colic. Additionally, a sub-aim of this study was to evaluate PF total nucleated cell counts (TNCC), total protein (TP), and lactate in the context of our population for agreement with the previous literature on their utility in colic diagnosis and prognostication. Our overarching hypothesis was that quantitative neutrophil values, TNCC, TP, and lactate in PF would be significantly different between different colic lesion types (including strangulating, non-strangulating, and inflammatory lesions), but not between different anatomic locations of colic lesions (including small or large intestine). Specifically, we hypothesized that both absolute and % PF neutrophil counts would be significantly elevated in horses with strangulating or inflammatory GI lesions compared to non-strangulating lesions. Additionally, we hypothesized that higher absolute and % PF neutrophil counts would be associated with increased complications during hospitalization, including the development of SIRS or postoperative reflux (POR), and mortality. Finally, we hypothesized that PF TNCC, TP, and lactate would follow similar trends as these quantitative neutrophil values, being elevated in strangulating and inflammatory lesions versus non-strangulating lesions and higher in horses that developed postoperative complications and/or did not survive to discharge, as has been noted in the previous literature [10,11,12,13].

## 2. Materials and Methods

Medical records between the years of 2010–2020 were searched for horses presenting to the University of Georgia for the evaluation of colic and on which an abdominocentesis and abdominal fluid cytology was performed. Only the first abdominocentesis sample with accompanying cytology was evaluated in horses where more than one sample was taken. Horses were excluded if the qualifying abdominocentesis was performed more than 48 h after admission or was obtained within 30 days post-abdominal exploratory surgery. Horses were also excluded if no systemic complete blood count was performed on admission, the final diagnosis was not related to the GI tract or diffuse abdominal disease (which included idiopathic septic peritonitis or primary peritonitis), the horse had peritonitis secondary to a ruptured GI viscus or intra-abdominal mass or abscess, or if there was insufficient information in the medical record to categorize the lesion localization or type. Due to variability in reporting the administration of medications prior to presentation, the administration of drugs such as NSAIDs prior to abdominocentesis was not considered an exclusion factor for this study.

Information collected from the medical record included the duration of colic prior to admission (classified as either <12 h, 12–24 h, or >24 h), physical exam parameters on admission and throughout hospitalization including rectal temperature, pulse, respiratory rate (the frequency of collection was determined by the attending clinician), clinicopathologic parameters (systemic bloodwork and PF evaluation), lesion localization (small intestine, large intestine, both small and large intestine, or peritoneum including peritonitis and hemoabdomen), and lesion type (non-strangulating, strangulating, inflammatory enteropathy, or peritonitis). The PF clinicopathologic data analyzed included PF total nucleated cell count (PF TNCC), % and absolute neutrophil count, PF total protein (PF TP), and PF lactate. Systemic laboratory data analyzed included white blood cell (WBC) count, % and absolute neutrophil count, packed cell volume (PCV), total protein (TP), and lactate. Lesion type and site was determined by one of the following: the final diagnosis as noted at surgery or on necropsy for those horses having those procedures or for horses that received medical management only, the diagnosis of non-strangulating, strangulating, or inflammatory disease, and the site of the lesion that was assigned by the attending clinician, utilizing a combination of systemic and PF clinicopathologic parameters, diagnostic imaging, and clinical presentation. Inflammatory lesions included colitis, enteritis, or enterocolitis as diagnosed by the attending clinician via surgery or necropsy, or using a combination of diagnostic parameters including the ultrasonographic evidence of intestinal thickening, the histopathology of intestinal or rectal biopsies, and characteristic bloodwork changes such as leukopenia or hypoproteinemia, distinguishing clinical signs such as fever, diarrhea or profuse nasogastric reflux in the absence of a strangulating lesion, and/or culture of diarrhea or nasogastric reflux. Peritonitis cases were defined as horses reaching a PF TNCC of over 10,000 cells/μL during the duration of their hospitalization and treatment, and exclusion from other GI categories [14,15]. These horses were assigned a lesion site of “peritoneum” and a lesion type of “peritonitis” to distinguish them from other GI lesion categories.

The collected hospitalization information included the type of management (surgical, medical, or no treatment), the development of complications during hospitalization (SIRS, or POR if they received abdominal exploratory surgery), and survival to discharge. Horses were considered to have received “no treatment” if they were euthanized with no further treatment beyond initial rapid stabilization, including intravenous medications for pain or sedation to facilitate admission evaluation (such as flunixin meglumine or an alpha-2 agonist) or an intravenous fluid bolus. These horses were not assigned information on the development of hospitalization complications. Horses were determined to have developed SIRS during hospitalization if they had at least 2 of the following factors simultaneously following admission: a heart rate greater than 60 beats per minute, a respiratory rate greater than 30 breaths per minute, a rectal temperature above 101.5 °F [38.6 °C], or a white blood cell (WBC) count greater than 12,500 cells/μL or less than 4500 cells/μL with >10% band neutrophils, one of which had to be either an abnormal rectal temperature or abnormal leukocyte count (adapted from the neonatal SIRS criteria established by Wong et al. 2018 [16]). While these criteria were established in foals, the authors felt that utilizing these stricter criteria in adult horses minimized the classification of horses with SIRS if tachycardia and tachypnea were the only two criteria, which can commonly be caused by pain alone. This is also supported in a study evaluating adult horses with a new SIRS scoring system, which demonstrated that horses with ≥3 SIRS criteria (thus including either abnormal rectal temperature or WBC count) had increased odds of death compared to horses with ≤2 SIRS criteria [10]. Horses were considered to have POR if they produced greater than 2 L of gastric reflux any time postoperatively [17]. Horses were considered to survive to discharge if they were noted to be discharged from the hospital during that specific admission. Horses that died or were euthanized during hospitalization, regardless of whether this was for prognosis or financial reasons, were considered to have not survived to discharge.

Quantitative parameters were assessed for normality by D’Agostino Pearson test. PF TNCC, % neutrophil count, total neutrophil count, TP, and lactate were analyzed for differences and associations with the site of lesion, lesion type, the development of SIRS during hospitalization, the development of postoperative reflux (if the patient received surgery), and survival to discharge using either Mann–Whitney rank sum analysis or Kruskal–Wallis Test with Dunn’s correction for multiple comparisons. Additionally, the ratios of PF-to-systemic TNCC, neutrophil counts, TP, and lactate were compared between survivors and non-survivors by rank sum test. Parameters that were found to be significantly different between survivors and non-survivors were entered into a stepwise, multivariable logistic regression analysis for predicting survival to discharge. For parameters significant in the multivariable logistic regression analysis, a receiver–operator curve analysis was performed to evaluate sensitivity and specificity metrics for survival. Where applicable, data are presented as median (range) or box whisker IQR figures. Statistical significance was set at *p* < 0.05. Statistical analyses were performed with two software packages (MedCalc Version 22 by MedCalc Software Ltd., Ostend, Belgium and Prism Version 10 by GraphPad Software, LLC., Boston, MA, USA)

## 3. Results

### 3.1. Descriptive Data

During the study period, there were 971 horses that had abdominal fluid cytology results recorded in the medical record. After removing cases related to exclusion criteria, the final study population included 342 horses for analysis. Of these horses, 196 were geldings, 132 were mares, and 14 were stallions. Ages ranged from 3 months to 28 years, with a median age of 13 years. Breeds included 103 stock-type horses (quarter horse, paint, appaloosa, or appendix), 48 Thoroughbred/Irish Sport Horses, 57 Warmbloods/Andalusians, 16 Tennessee Walking Horses, 12 Saddlebreds, 24 draft/draft-cross or Friesians, 15 ponies, 6 miniature horses, 29 Arabians, 3 donkeys/mules, and 29 horses from other breeds.

A total of 185 horses were diagnosed with small intestinal lesions, of which 70 horses were diagnosed with non-strangulating lesions, 80 horses were diagnosed with strangulating lesions, and 35 horses were diagnosed with inflammatory lesions. A total of 128 horses were diagnosed with large intestinal lesions, of which 73 horses were diagnosed with non-strangulating lesions, 8 horses were diagnosed with strangulating lesions, and 47 horses were diagnosed with inflammatory lesions. Nineteen horses were diagnosed with lesions affecting both the small and large intestine, all of which were inflammatory lesions. Ten horses were diagnosed with disease affecting the peritoneum, and all ten cases were considered as idiopathic peritonitis.

A total of 137 horses underwent abdominal exploratory surgery, 192 underwent medical therapy, and 13 horses were euthanized shortly after admission with no therapy initiated beyond immediate stabilization to facilitate admission examination. Of the horses undergoing colic surgery, 84 horses survived to discharge, and 53 horses did not survive to discharge, including 24 horses that were euthanized on the table during surgery and thus were not included in postoperative complications analysis. Of those that underwent and recovered from surgery, 93 horses did not develop SIRS while 20 horses did develop SIRS during hospitalization. Additionally, 72 of these horses did not develop POR while 41 horses did develop POR. Of the horses undergoing medical management, 170 horses survived to discharge, while 22 horses did not survive to discharge. Of the medically managed horses, 167 did not develop SIRS during hospitalization while 25 horses did develop SIRS during hospitalization.

### 3.2. Anatomic Site of Colic Lesion

When evaluating the anatomic site of colic lesions, PF TNCC, absolute neutrophil count, and % neutrophils did not differ between small versus large intestinal lesions. However, horses diagnosed with the disease of the peritoneum (idiopathic peritonitis) had significantly higher TNCC (*p* < 0.0001), total neutrophil count (*p* = 0.001), and % neutrophils (*p* = 0.003) than horses with either small or large intestinal lesions. Additionally, TNCC was higher in horses with peritoneal disease than horses with lesions affecting both the small and large intestine (*p* < 0.0001). Horses with large intestinal lesions had significantly lower TP within the PF than those with small intestinal lesions (*p* < 0.0001), lesions affecting the small and large intestine, and horses with a disease of the peritoneum (*p* < 0.0001). Finally, horses with small intestinal lesions had significantly higher PF lactate than those with large intestinal lesions (*p* = 0.019) (Table 1).

### 3.3. Colic Lesion Type

When lesion type was considered, horses with peritonitis had higher TNCC (*p* < 0.0001), total neutrophil count (*p* < 0.0001), and % neutrophils (*p* < 0.0001) within the PF than horses with strangulating, non-strangulating, or inflammatory lesions. Additionally, horses with inflammatory GI lesions had higher TNCC than horses with non-strangulating lesions (*p* < 0.0001); however, there was no difference in TNCC between inflammatory and strangulating lesions. Horses with strangulating lesions had significantly higher % neutrophils (0.003), neutrophil counts (*p* = 0.026), and TNCC (*p* = 0.013) compared to non-strangulating lesions. However, there was no difference between % neutrophils or neutrophil count in inflammatory versus strangulating lesions. TP was noted to be significantly lower in horses with non-strangulating lesions versus horses with strangulating lesions or peritonitis (*p* < 0.0001). Additionally, horses with inflammatory lesions had significantly lower TP than strangulating lesions (*p* = 0.003), but there was no difference between horses with inflammatory versus non-strangulating lesions. Finally, lactate was significantly lower in non-strangulating lesions than horses with strangulating lesions (*p* = 0.001, Table 2).

### 3.4. Complications

Horses who developed SIRS during hospitalization had significantly higher PF TNCC (*p* = 0.048), total neutrophil count (*p* = 0.040), TP (*p* = 0.014), and lactate (*p* = 0.022) compared to horses that did not develop SIRS during hospitalization. There was no difference in % neutrophil count between these groups.

Horses who developed POR had significantly higher PF TNCC (*p* = 0.010), higher total neutrophil counts, (*p* = 0.011), higher TP (*p* = 0.0001), and higher lactate (*p* = 0.003) on admission compared to horses that did not develop POR (Table 3). There was no difference in % neutrophil count between these groups.

### 3.5. Survival to Discharge

Horses who did not survive to discharge had significantly higher PF total neutrophil counts (*p* < 0.0001), % neutrophils (*p* < 0.001), TP (*p* < 0.001), and lactate (*p* = 0.003) compared to horses that survived to discharge. Ratios between PF and systemic TP was found to be significantly higher in horses that did not survive versus those that did survive to discharge (*p* <0.001, Table 4). Ratios between PF and systemic TNCC, % neutrophils, total neutrophils, and lactate were not significantly different between survival groups. Significant parameters from univariable analysis (PF total neutrophil counts, % neutrophils, TP, lactate, and ratio of PF-to-systemic TP) were entered into a multivariable logistic regression stepwise model, and only the PF-to-systemic total protein ratio remained predictive for survival (odds ratio 488.6, 95% CI of OR 2.4–97,993.5; *p* = 0.022). A receiver–operator curve analysis for the fluid-to-systemic total protein ratio for predicting non-survival had AUC = 0.693 (*p* < 0.003); a ratio threshold of >0.55 had a sensitivity of 54%, and a specificity of 78% for not surviving to hospital discharge.

## 4. Discussion

Neutrophils accumulate within GI tissues during states of inflammation and ischemia, and as tissue vasculature becomes compromised, these cells begin to leak into the peritoneal cavity [18]. We hypothesized that this phenomenon occurs to varying degrees based on the severity of a GI compromise, but is not dependent on the site of the lesion. Thus, we predicted that quantitative PF neutrophil values such as % neutrophils and total neutrophil count could differentiate types of colic lesions. The data from this study supported the overarching hypothesis that quantitative PF neutrophil values would be different between different GI lesion types, but not anatomic location (Table 1 and Table 2). Our data did not support our more specific assertion that these PF neutrophil values would stratify all GI lesion types (Table 2). Furthermore, our data supported the hypothesis that increased PF neutrophil values would be associated with increased complications, as there were significantly increased PF total neutrophil counts in horses that developed SIRS or POR versus those that did not (Table 3). Additionally, increased % PF neutrophils and total neutrophil counts were associated with non-survival in colic horses in a univariable analysis, but did not remain associated with survival in a multivariable logistic regression analysis (Table 4).

Strangulating gastrointestinal lesions had a significantly higher PF TNCC, total neutrophil count, and % PF neutrophils compared to non-strangulating GI lesions; however, there was no difference between strangulating and inflammatory lesions. This suggests that quantitative PF neutrophil values are unable to discriminate strangulating versus inflammatory lesions, and as sole parameters, are unlikely to be an indicator of the need for surgical intervention. This is likely due to the fact neutrophils are involved in the pathophysiology of both ischemic and inflammatory intestinal lesions [19,20] and must be interpreted in light of other PF parameters. This includes TP, which was significantly higher in strangulating lesions than inflammatory or non-strangulating obstructions. This is supported in a study by Matthews et al. who found PF TP had a moderate sensitivity and specificity for the need for abdominal surgery, which is almost invariably required for strangulating lesions [2]. Interestingly, PF lactate was only significantly different between strangulating and non-strangulating lesions, similar to previous studies [11], but not between strangulating and inflammatory lesions. This was also demonstrated previously in a retrospective study by Shearer et al. 2018, who suggested that not only does bowel ischemia increase PF lactate, but that bowel distention and vascular compromise caused by inflammation may also contribute to lactate accumulation [21]. Thus, PF lactate must also be interpreted in conjunction with other PF parameters such as total protein and % PF neutrophils.

This study suggests that normalized PF neutrophil values (such as % PF neutrophils) may not add value to our diagnostic abilities compared to absolute PF cell counts. Both % PF neutrophils and total PF neutrophils were different between strangulating and non-strangulating gastrointestinal lesions. Additionally, absolute PF TNCC was significantly different between inflammatory and non-strangulating GI lesions and between strangulating and non-strangulating lesions. However, there was no difference between PF TNCC, % neutrophils, or total neutrophils in inflammatory versus strangulating lesions, which is often a more difficult differentiation and is critical for determining if surgical intervention or medical management is needed. This is consistent with previous studies, which found that PF TNCC had low positive and negative predictive values for determining the need for surgery (67% and 67%, respectively) [2]. The inability of both normalized and absolute leukocyte values to differentiate strangulating lesions from all other lesion types is likely due to the wide range and overlap in leukocyte values seen between horses with different lesion types. This large overlap likely stems from similar underlying pathologies between lesion types, particularly with increased duration and severity. Strangulating lesions elicit acute inflammation and leukocyte accumulation through ischemic mechanisms, and the degree of tissue damage often appears clinically more severe than that caused by non-strangulating lesions. This leads to a release of damage-associated-molecular patterns (DAMPs), which are a major signal for neutrophil influx [22]. This is supported in human studies which have demonstrated increased neutrophil-to-lymphocyte ratios that are seen systemically in patients with acute mesenteric ischemic lesions compared to other causes of acute abdominal disease [23]. In contrast, non-strangulating lesions elicit inflammation due to GI distention, which tends to elicit less severe tissue damage until the lesion becomes more chronic; this was supported in an equine model where non-strangulating lesions caused only a mild increase in neutrophil accumulation within GI tissues [24] in contrast to ischemic lesions [19]. It is also possible that less severe and more chronic GI injury may lead to less DAMP accumulation and vascular damage, and thus fewer infiltrating neutrophils; this may explain the difference in PF TNCC, total neutrophils, and % neutrophils between strangulating and non-strangulating lesions. However, diffuse inflammatory lesions can also elicit profound neutrophilic influx [25], which as demonstrated here, can lead to increases in PF neutrophil counts that are similar to strangulating lesions. Inflammatory gastrointestinal diseases can be caused by bacteria such as *Clostridium difficile* or *Salmonella enterica* which contain known pathogen-associated molecular patterns (PAMPs) that elicit neutrophilic influx [25,26,27]. Thus, similarities in the underlying inflammatory pathophysiology of strangulating and inflammatory GI lesions likely explain why quantitative PF neutrophil values were unable to discriminate between these lesion types.

As hypothesized, no association was identified between quantitative PF neutrophil values and lesion location. This is likely due to similar underlying pathologic mechanisms of tissue injury involving neutrophils in the small and large intestine [19,28]. The only parameters evaluated that were associated with lesion site were PF TP and PF lactate, both of which were significantly higher in horses with small intestinal lesions compared to large intestinal lesions. This finding should be interpreted cautiously, as it likely reflects the study population rather than the lesion site. This population contained a higher proportion of strangulating lesions within the small intestinal group (approximately 43% of all small intestinal lesions were considered strangulating) compared to the large intestinal group (of which only approximately 6% were considered strangulating). As previously discussed, strangulating lesions were shown to have higher PF TP and PF lactate values compared to other lesion types, which likely explains the difference in these parameters between lesion sites.

Additionally, as hypothesized, elevated PF neutrophil values were associated with an increased risk of specific complications including SIRS and POR. Both PF TNCC and total neutrophil counts were increased in the abdominal fluid of horses who developed POR and SIRS, suggesting a potential role for neutrophils within the pathology of both disease processes. In addition, PF TP and lactate were also elevated in both disease states, which likely reflects the severity of these lesions. It is possible that increased neutrophil counts are present with more severe GI lesions and may play a role in the development of complications by directly eliciting or exacerbating tissue damage; however, this could not be directly evaluated in this retrospective study.

In horses, gastric reflux following colic surgery can be indicative of several issues including GI stricture formation, the presence of adhesions, anastomotic complications, or is often due to GI ileus, or stasis. While the pathophysiology of ileus in horses remains ill-defined, several studies suggest that GI inflammation following different types of intestinal trauma, including ischemia/reperfusion and surgical manipulation, may play a role in GI motility disturbances. Studies of ischemic equine bowels have demonstrated that neutrophils accumulate within affected tissues during both the ischemia and reperfusion phases [19,29], as well as within serosa proximal to the ischemic lesion [4]. Additional studies in rodents have demonstrated that surgical manipulation leads to increased neutrophil accumulation that coincides with impaired GI smooth muscle contraction [7,30], and that inhibiting leukocyte migration into these tissues alleviated postoperative ileus [7]. Taken together, these previous studies suggest that neutrophilic inflammation is likely involved in the development of GI ileus and that neutrophil-targeting therapies may be useful for the treatment of ileus and POR in horses. This could include the therapeutic targeting of neutrophil extracellular traps (NETs) which are known to contribute to ischemia–reperfusion and intestinal disease pathology by enhancing thrombosis, perpetuating inflammation, and eliciting direct cell damage [31]. Studies in mice have demonstrated that the administration of DNase1, which targeted the extracellular DNA components of NETs, led to decreased intestinal tissue injury and decreased neutrophil infiltration into the GI tissues [31,32]. Clinical trials have invested the use of DNase1 in humans for the treatment of cystic fibrosis [33], among other diseases, and thus these drugs may be clinically available for other species in the future.

Our data demonstrated increased neutrophil values in the PF of horses prior to surgery who ultimately developed POR, which we hypothesize is in part reflective of increased neutrophilic inflammation and vascular injury within the gut from their inciting lesion (such as was seen in strangulating lesions, Table 2). Thus, it is also possible that neutrophil-targeting strategies prior to surgery might also be useful as a prophylactic for the development of POR. It is worth noting that this study aimed to determine predictors and prognosticators of POR, which is a more common cause of significant morbidity and mortality in our surgical population (37.4%) versus horses that refluxed while undergoing medical management (12.8%). Thus, the effect of PF parameters on reflux were not evaluated in medically managed horses in our study.

The severity of disease (such as length of compromised bowel, duration of disease, etc.), as evidenced by the relationship between TP and lactate with development of SIRS and POR, likely also plays a role in the development of complications above what is caused by neutrophilic influx alone; this is likely influenced by inflammation systemically and within the abdominal cavity to exacerbate SIRS and ileus. PF TP and lactate were both significantly elevated in horses with strangulating lesions, as has been shown previously [9,11], and horses with strangulating lesions are more likely to develop SIRS [10]. Studies have demonstrated that systemic lactate is correlated with the presence of SIRS in neonates [34,35]. Additionally, studies in adult horses have shown that combining systemic lactate with SIRS scoring can provide a more accurate method of predicting survival [10]. While we did not evaluate the relationship between systemic lactate and development of SIRS, PF lactate is also a valuable marker to evaluate because this value changes more acutely than systemic lactate in the event of GI disease. Peloso, et al. 2012 noted a significant difference between PF lactate in strangulating lesions versus non-strangulating lesions on admission, but systemic lactate did not significantly differ between groups at admission [36]. However, ultimately, with some chronicity, changes within the abdomen influence systemic status and can lead to the development of SIRS in colic cases. Our ability to differentiate the influence of these different parameters (quantitative neutrophil values, TP, and lactate) when disease severity is considered is an area of future study.

A univariable model for survival, defined as survival to discharge from the hospital, was created that included the following PF clinicopathologic parameters: TNCC, % neutrophils, absolute neutrophil count, lactate, and TP. Additionally, ratios of PF-to-systemic values for each of the aforementioned parameters were also included, as PF parameters often become elevated more rapidly than systemic parameters in cases of GI disease [1]. Comparing the difference between these values has been shown to help predict surgical necessity in horses [11]. In this model, PF total neutrophil count, PF % neutrophils, PF TP, PF lactate, and the ratio of PF-to-systemic TP were all significantly higher in horses that did not survive to discharge versus those that did. When these parameters were entered into a multivariable logistic regression stepwise model for survival, the only parameter that was included in the final model that was predictive of survival was the ratio of PF-to-systemic TP. Previous studies have also utilized linear regression modeling to demonstrate that both PF TP and systemic TP can predict survival. Furr et al. demonstrated that elevated PF TP can predict survival and was utilized as part of a predictive model that had a sensitivity of about 53% and a specificity of about 90% [12]. In our study, PF-to-systemic TP with a ratio threshold of >0.55 (PF TP above about one-half that of the systemic TP) had a sensitivity of 54% and a specificity of 78% for non-survival, which is similarly moderately specific to the model developed by Furr et al. Interestingly, two previous studies determined that decreasing systemic TP was associated with an increased risk of non-survival [17,37]. This is likely also reflected in our PF-to-systemic ratio, as a higher ratio was predictive of non-survival while PF TP alone was not as was seen in Furr et al. 1995. To the author’s knowledge, this is the first time that this ratio has been evaluated and shown to be correlated with survival.

Interestingly, PF lactate has been shown previously to be significantly increased in horses that did not survive to discharge [10,11]. This is similar to the current study; however, this did not remain significant in our multivariable model. It is possible that regional differences in the lesion types seen or colic duration could explain this disparity [32]. Another previous study demonstrated that both PF TNCC and total neutrophil counts were higher in horses that were taken to surgery but did not survive compared to horses managed medically; however, there was no difference between horses that were taken to surgery and died versus those that survived surgery, or compared to horses that were euthanized prior to surgical intervention, and thus these data are difficult to interpret [13]. Additionally, this study did not utilize a logistic regression model and thus is difficult to compare to our study.

There are several limitations to this study due to the retrospective nature of the analysis. Firstly, we were not able to differentiate between reasons for euthanasia and ultimate non-survival. The decision to euthanize is a complex one that involves several different and often overlapping factors, including poor prognosis, financial limitations, or both, and may bias retrospective survival studies [38]. A further limitation of this study includes the use of clinical diagnosis of records for the categorization of some medically treated horses. Many of these diagnoses were confirmed via necropsy, histopathology (such as rectal or jejunal biopsy), or a culture of nasogastric reflux or diarrhea. However, for others without a definitive diagnosis using those means, the diagnosis assigned by the attending clinician was utilized. This was based on characteristic diagnostic findings, including ultrasonographic imaging, characteristic bloodwork changes, and accompanying clinical signs such as the presence or absence of fever, nasogastric reflux, or diarrhea. While it is possible that lesions were miscategorized by the admitting clinician, their presentation, case progression, and management fit best with other horses within the category in which they were included, as deemed appropriate by the admitting clinician. Another limitation of the retrospective nature of this study includes the exact timing of blood collection for CBC and accompanying physical exam to determine SIRS status during hospitalization. If leukocyte values were one of the criteria for SIRS, the physical exam values noted most closely to the time the CBC was reported in the medical record was used to categorize for SIRS. Horses routinely received physical exams every 6 h while in hospital for colic treatment, but because the exact time of blood collection for CBC in accordance with their physical exam was not specified in the record, this could potentially have led to a misclassification of some horses and should be considered when interpreting these data.

Additional limitations to this study include low study numbers within certain groups of horses. This led us to separate horses into the broader categories of lesion site (small intestine, large intestine, both small and large intestine, or peritoneum) or lesion type (strangulating, non-strangulating, inflammatory, or peritonitis). This allocation may have confounded some of the lesion site data, as most strangulating lesions (which were shown to have higher PF TP and lactate values) were small intestinal cases. This may have influenced the finding that small intestinal lesions also had higher PF TP and lactate values than did large intestinal lesions and should be considered when interpreting these data. Multicenter studies evaluating a greater number of horses could determine if data from multiple populations may improve our ability to predict certain outcomes in different populations. Finally, it must also be noted that the median TNCC and neutrophil counts that were associated with specific outcomes, such as the development of complications or survival, remained within the upper end of some normal reference ranges [1]. Therefore, these values should still be considered with other clinical and clinicopathologic parameters (such as PF TP or % neutrophils, depending on the outcome) to determine their utility within the larger clinical picture. Monitoring changes in these values in sequential abdominocentesis over time might also prove to be a useful measure and warrants further study.

## 5. Conclusions

This study adds new insight to the utility of PF clinicopathologic parameters for diagnosis and predicting postoperative outcomes in equine colic patients. Increased PF neutrophil counts, % neutrophils, TNCC, TP, and lactate were all associated with the presence of strangulating lesions; however, only TP was able to differentiate strangulating from inflammatory lesions. Additionally, increased PF neutrophil counts, TNCC, TP, and lactate, but not % PF neutrophils, were associated with the development of significant complications, including SIRS and POR. However, only the ratio of PF-to-systemic TP ratio was able to predict survival in a multivariate analysis. This suggests that while quantitative PF neutrophil values may help to support the diagnosis of strangulating lesions and the development of complications, they were not indicative of survival. Future prospective studies are warranted to investigate the ability of PF-to-systemic TP ratio to predict survival in different colic populations.

## Figures and Tables

**Table 1:** Comparison of peritoneal fluid values between anatomic site of colic lesions.

Peritoneal Fluid Value	Small Intestine (n = 185)	Large Intestine (n = 128)	Small and Large Intestine (n = 19)	Peritoneum (n = 10)	*p* Value
TNCC (cells/μL)	2200 (300–82,500) ^a^	1300 (100–112,620) ^a^	1800 (300–34,600) ^a^	112,900 (700–625,000) ^b^	<0.0001
Neutrophil Count (cells/μL)	1083 (0–74,250) ^a^	675 (0–50,424) ^a^	794 (18–30,448) ^a^	104,209 (840–269,670) ^b^	0.001
% Neutrophils	64 (0–99) ^a^	60 (0–97) ^a^	52 (6–97) ^a^	90.5 (70–98) ^b^	0.0026
Total Protein (g/dL)	3.2 (0.3–9.0) ^a^	2.0 (0.2–4.8) ^b^	3.2 (0.7–5.5) ^a^	4.0 (1.6–5.9) ^a^	<0.0001
Lactate (mmol/L)	4.6 (0.8–22.0) ^a^	2.6 (0.8–21.8) ^b^	3.8 (0.9–11.0) ^ab^	4.2 (0.7–6.3) ^ab^	0.0019

Data are displayed as median and range values. Within a row, medians that do not have a common superscript (a or b) are statistically different.

**Table 2:** Comparison of peritoneal fluid values between colic lesion types.

Peritoneal Fluid Value	Non-Strangulating (n = 143)	Strangulating (n = 88)	Inflammatory (n = 101)	Peritonitis (n = 10)	*p* Value
TNCC (cells/μL)	1200 (100–36,800) ^a^	2300 (300–82,500) ^c^	2800 (300–112,620) ^c^	112,900 (700–625,000) ^b^	<0.0001
Neutrophil Count (cells/μL)	650 (0–27,606) ^a^	1508 (0–74,250) ^c^	1020 (0–50,424) ^ac^	104,209 (15–269,670) ^b^	<0.0001
% Neutrophils	58 (0–99) ^a^	72 (0–99) ^b^	60 (1–97) ^ab^	91 (70–98) ^b^	<0.0001
Total Protein (g/dL)	2.3 (0.2–5.9) ^a^	3.7 (1.0–6.6) ^b^	2.5 (0.4–9) ^ac^	4.0 (1.6–5.9) ^bc^	<0.0001
Lactate (mmol/L)	3.1 (0.8–16.1) ^a^	5.3 (0.8–22) ^b^	4.0 (0.9–16.0) ^ab^	4.2 (0.7–6.3) ^ab^	0.0014

Data are displayed as median and range values; *p*-value is for overall comparisons. Within a row, medians that do not have a common superscript (a, b, or c) are statistically different.

**Table 3:** Analysis of peritoneal fluid clinicopathologic values and development of systemic inflammatory response syndrome (SIRS) and postoperative reflux (POR) during hospitalization.

Peritoneal Fluid Value	SIRS (n = 45)	No SIRS (n = 260)	*p* Value, SIRS vs. No SIRS	POR (n = 41)	No POR (n = 72)	*p* Value, POR vs. No POR
TNCC (cells/μL)	2800 (400–243,500)	1500 (0–625,000)	0.048	2500 (0–75,300)	1400 (0–112,620)	0.010
Neutrophil Count (cells/μL)	1184 (0–219,150)	797 (0–269,670)	0.040	1800 (0–70,782)	750 (0–74,011)	0.011
% Neutrophils	70 (0–97)	61 (0–99)	0.085	70 (0–95)	58 (0–97)	0.061
Total Protein (g/dL)	3.4 (0.7–6.6)	2.5 (0.2–9.0)	0.014	3.8 (1.0–6.6)	2.6 (0.5–5.1)	0.0001
Lactate (mmol/L)	5.6 (1.1–18.0)	3.9 (0.7–20.6)	0.022	6.0 (1.1–20.6)	3.7 (0.8–14.6)	0.003

Data are displayed as median and range values.

**Table 4:** Analysis of peritoneal fluid clinicopathologic values and PF to systemic clinicopathologic ratios and survival to discharge.

Peritoneal Fluid Value	Survivors (n = 254)	Non-Survivors (n = 88)	*p* Value
TNCC (cells/μL)	1500 (0–625,000)	2700 (100–303,000)	0.083
Neutrophil Count (cells/μL)	799 (0–219,150)	1517 (0–269,670)	0.012
% Neutrophils	60 (0–99)	78 (0–99)	0.0005
Total Protein (g/dL)	2.5 (0.2–9.0)	3.4 (0.7–6.6)	<0.001
Lactate (mmol/L)	3.9 (0.7–16.1)	5.3 (0.8–22.0)	0.007
**Peritoneal Fluid: Systemic Ratios**			
TNCC	0.22 (0.01–67.20)	0.38 (0.02–64.47)	0.170
Neutrophil Count	0.23 (0.01–16.42)	0.36 (0.01–345.50)	0.23
% Neutrophils	0.87 (0.01–3.72)	0.99 (0.01–14.17)	0.15
Total Protein (g/dL)	0.36 (0.01–1.37)	0.53 (0.08–1.00)	<0.0001
Lactate	1.46 (0.35–10.00)	1.58 (0.01–11.50)	0.16

Data are displayed as median and range values.

## Data Availability

All data are contained within the article.
